# Inpatient Hemato-Oncology Service Use Before, During, and After COVID-19 Restrictions in a Romanian Tertiary Center

**DOI:** 10.7759/cureus.112380

**Published:** 2026-07-09

**Authors:** Paul C Toboltoc

**Affiliations:** 1 Pathology, Sibiu County Emergency Clinical Hospital, Sibiu, ROU

**Keywords:** covid-19, healthcare utilization, hematologic malignancies, hospital days, hospitalizations, inpatient care, pandemic, romania

## Abstract

Introduction

The coronavirus disease 2019 (COVID-19) pandemic disrupted cancer diagnosis and treatment pathways, but hospital-based evidence on inpatient hemato-oncology service use in Eastern Europe remains limited. This study evaluated how the pandemic period affected case capture, diagnostic confirmation, repeated inpatient representation, hospital admissions, and hospital days among adults with lymphoma, leukemia, or multiple myeloma treated in a Romanian tertiary center.

Materials and methods

A retrospective observational single-center analysis was performed at Sibiu County Emergency Clinical Hospital, Sibiu, Romania. Adult patients identified in hospital records between March 11, 2018, and March 8, 2024, were grouped into pre-pandemic, pandemic, and post-restriction periods. The analysis followed three cohort layers: initially identified patients, diagnostically confirmed cases, and final longitudinally eligible patients, defined as diagnostically confirmed patients with at least two standard inpatient admissions in the corresponding study period. Population-normalized hospital-based rates, rate ratios (RRs), 95% confidence intervals (CIs), proportion tests, chi-square tests, non-parametric comparisons, sensitivity analyses by major disease group, and adjusted patient-level models were used.

Results

The initially identified patient cohort included 1,314 patients, of whom 1,188 were diagnostically confirmed, and 749 met final longitudinal eligibility criteria. All patient counts decreased numerically during the pandemic period, but these changes did not reach statistical significance. In contrast, inpatient service-use indicators showed significant contraction. Hospitalizations declined from 1,450 before the pandemic to 1,325 during the pandemic period (RR 0.91, 95% CI 0.85-0.98, p = 0.015), while hospital days decreased from 8,123 to 6,553 (RR 0.81, 95% CI 0.78-0.83, p < 0.001). After restrictions, hospitalizations returned to baseline, but hospital days remained below the pre-pandemic level (RR 0.90, 95% CI 0.88-0.93, p < 0.001). Broad lymphoma, leukemia, and multiple myeloma distributions remained stable across periods. Disease-specific sensitivity analyses showed significant pandemic-period reductions in aggregate hospital days across all three major groups, whereas adjusted models showed comparable admissions and hospital days per patient retained in the final longitudinal cohort.

Conclusions

In this Romanian tertiary-center cohort, the pandemic period was associated mainly with aggregate inpatient service-use contraction rather than with broad disease-mix change. The findings indicate that stable cohort composition may coexist with reduced inpatient bed-day exposure. Combined monitoring of case capture, diagnostic confirmation, longitudinal inpatient representation, admissions, and hospital days may provide a descriptive hospital-based framework for distinguishing diagnostic-volume changes from service-intensity changes during healthcare disruptions.

## Introduction

The World Health Organization (WHO) characterized coronavirus disease 2019 (COVID-19) as a pandemic on March 11, 2020 [1], while Romania’s national state of alert was no longer extended after March 8, 2022 [2]. These dates define clinically and administratively relevant intervals for evaluating hospital activity before, during, and after the main pandemic restriction period. Across oncology services, the pandemic was associated with delayed diagnostic pathways, reduced diagnostic testing, treatment adaptation, and fewer recorded cancer diagnoses [3,4]. In patients with hematologic malignancies, early evidence also showed high COVID-19-related vulnerability, while European Society for Medical Oncology (ESMO)-European Hematology Association (EHA) consensus and practical management recommendations emphasized infection risk reduction, treatment prioritization, and preservation of clinically necessary hematology care during periods of system disruption [5-7].

Pandemic-period effects in hematologic malignancies may differ from the screening-driven declines described for many solid tumors. In the Swedish Lymphoma Register, fewer lymphoma diagnoses were recorded during March-June 2020, although annual lymphoma incidence in 2020 and 2021 remained close to the 2017-2019 reference period [8]. In Oxford, a hematology service reported 54% fewer new hematological malignancies diagnosed by histopathology during lockdown, indicating a marked reduction in specialist diagnostic throughput [9]. Multiple myeloma studies further described delayed diagnosis, more emergency presentations, fewer new diagnoses in 2020, and adverse survival signals among patients diagnosed during the pandemic [10,11]. Hemato-oncology service data from Kazakhstan also showed altered access to specialized care, including changes in emergency admissions, transfers, and mortality [12]. In classical Hodgkin lymphoma, a Turkish cohort reported a longer median symptom-to-diagnosis interval during the pandemic, together with a numerically higher proportion of advanced-stage disease [13].

The existing literature, including epidemiological studies, cancer registry analyses, and disease-specific hematology cohorts, has shown that the COVID-19 pandemic affected diagnostic pathways, cancer detection, and continuity of care [3,4,8-13]. At the hospital level, these disruptions can also be evaluated through operational indicators that describe routine care delivery, repeated admissions, and inpatient workload. In hemato-oncology, this is particularly relevant because diagnosis, repeated admissions, treatment monitoring, complications, and supportive care often remain concentrated within specialist hospital pathways.

The present study evaluated hospital-based service use among adults with lymphoma, leukemia, or multiple myeloma treated at a Romanian tertiary center providing adult hematology care for the county-level population. The analysis distinguished initially identified patients, diagnostically confirmed cases, and final longitudinally eligible patients. In this study, final longitudinal eligibility referred to diagnostically confirmed patients with at least two standard inpatient admissions within the local hospital pathway during the corresponding study period, a threshold used to define repeated local inpatient representation for longitudinal service-use analysis. By analyzing patient counts together with admissions and bed-day volume, the study assessed whether pandemic-period changes were expressed mainly through diagnostic volume changes, broad disease group composition, or the intensity and continuity of inpatient hematology care.

The study was based on the hypothesis that pandemic-period changes would be more evident in aggregate inpatient service-use indicators than in the broad lymphoma-leukemia-multiple myeloma distribution. A second hypothesis was that the transition from diagnostic confirmation to final longitudinal inpatient eligibility would provide additional information on repeated local inpatient representation within the hospital pathway.

## Materials and methods

Study design, setting, and period definitions

This retrospective observational single-center study was conducted at the Sibiu County Emergency Clinical Hospital, Sibiu, Romania. Potential cases were retrieved from the hospital’s electronic administrative and clinical records and were manually reconciled with the available diagnostic documentation. Standard inpatient admissions were defined as conventional inpatient hospitalizations recorded in the institutional inpatient database, with an admission date, discharge date, and hospital day count. Outpatient visits, day-hospital or day-case encounters, emergency-only contacts without conversion to inpatient admission, and purely administrative records that did not generate a standard inpatient episode were not counted as standard inpatient admissions. Only standard inpatient episodes were used for hospitalization and hospital day endpoints.

Study population

The study included patients aged ≥18 years with lymphoma, leukemia, or multiple myeloma identified in hospital records between March 11, 2018, and March 8, 2024. The study interval was divided into three administratively defined periods: a pre-pandemic period from March 11, 2018, to March 10, 2020, a pandemic period from March 11, 2020, to March 8, 2022, and a post-restriction period from March 9, 2022, to March 8, 2024. The starting date of the pandemic period was aligned with the WHO pandemic characterization [1], while the post-restriction period was aligned with the lifting of Romania’s national alert-state restrictions on March 9, 2022 [2].

Cohort assignment was done according to the date of the first standard inpatient admission recorded in the institutional hematology pathway during the study interval. Patient counts were therefore mutually exclusive across the pre-pandemic, pandemic, and post-restriction periods. Hospitalization events and hospital days were allocated according to the admission date of each standard inpatient episode. For inpatient episodes crossing a study-period boundary, all hospital days associated with that episode were allocated to the period defined by the admission date and were not split across adjacent calendar periods.

The Sibiu County Emergency Clinical Hospital represents the main county-level tertiary hospital pathway for adult hemato-oncology care in Sibiu County. County-level population denominators were therefore used to calculate hospital-based rates per 100,000 person-years. These indicators were interpreted as population-normalized hospital case-capture and service-use rates, not as true population-based incidence estimates. Population denominators were obtained from the Romanian National Institute of Statistics TEMPO-Online resident-population series on July 1st for Sibiu County [14]. County denominators were used only to contextualize hospital-based workload within the hospital’s usual administrative catchment area.

Cohort layers and eligibility logic

The study was structured into three analytical cohort layers: 

Initially Identified Patients

All unique adult patients identified through electronic and administrative hospital extraction as potential lymphoma, leukemia, or multiple myeloma cases were termed the initially identified patients.

Diagnostically Confirmed Cohort

The diagnostically confirmed patients in the raw list included those patients who were confirmed as having a definite hematologic malignancy from available hospital documentation. Diagnostic confirmation required concordance between the extracted diagnostic label or keyword-based record and available clinical or diagnostic documentation, such as hematology discharge diagnoses, histopathology or cytology reports, bone marrow documentation, immunophenotyping reports, or other specialist hematology records. This reconciliation separated definite hematologic malignancies from uncertain, refuted, alternative, duplicated, or administratively generated records. Manual reconciliation was performed at the patient level by the author using deidentified institutional records and the predefined cohort-layer logic described above. The reconciliation process checked duplicate identifiers, conflicting inclusion or exclusion labels, alternative non-hematologic diagnoses, refuted or uncertain hematologic malignancy diagnoses, and records generated only by administrative or electronic extraction error; 

Longitudinally Eligible Cohort

The final longitudinally eligible cohort included diagnostically confirmed patients with at least two standard inpatient admissions within the corresponding study period. This threshold was used only to define repeated local inpatient representation for longitudinal service-use analysis and was not used as a criterion for diagnostic confirmation. Diagnostically confirmed patients who did not meet this threshold were retained as an attrition layer, because they may still represent clinically relevant patients managed mainly outside the inpatient pathway, transferred early, followed elsewhere, or deceased after a single admission. These patients were therefore not considered false cases, but confirmed cases without sufficient repeated local inpatient representation for the final longitudinal service-use cohort.

Patients were excluded from the final longitudinal cohort if they had fewer than two standard inpatient admissions, had continued care outside the local hospital pathway, had an alternative non-hematologic diagnosis, had an unconfirmed or refuted hematologic malignancy diagnosis, or were generated by administrative or electronic extraction error. Reconciliation flags were retained to allow manual audit of duplicate identifiers, conflicting inclusion/exclusion labels, and records present in the final dataset but absent from the inclusion/exclusion files. 

Variables and outcomes

The main study outcomes were initially identified patients, diagnostically confirmed cases, final longitudinally eligible patients, hospitalizations, and hospital days in each study period. Additional outcomes included final eligibility and attrition proportions, exclusion structure, disease group distribution, disease-specific final eligible rates, and patient-level measures, including age at first admission, admissions per patient, total hospital days per patient, and mean length of stay per patient.

The main disease groups were lymphoma, leukemia, and multiple myeloma. Analyses were performed at the broad disease-group level because the study focused on hospital-based case capture, attrition, and inpatient burden across major hematologic malignancy categories rather than on subtype-specific staging, treatment, or immunohistochemical phenotype.

Population-normalized indicators and statistical analysis

Annualized hospital-based rates were calculated per 100,000 county inhabitants using person-years for each study interval. County-level person-years were derived from the Romanian National Institute of Statistics TEMPO-Online resident-population series on July 1st, using the mid-year populations corresponding to 2018-2019, 2020-2021, and 2022-2023 for the pre-pandemic, pandemic, and post-restriction intervals, respectively.

Rate ratios (RRs) were used to compare the pandemic and post-restriction periods with the pre-pandemic reference period for count-based indicators, including initially identified patients, diagnostically confirmed cases, final longitudinally eligible patients, hospitalizations, hospital days, and disease-specific service-use indicators. RRs were calculated as the period-specific rate per population person-years divided by the corresponding pre-pandemic rate per population person-years. The 95% confidence intervals (CIs) for RRs were calculated on the logarithmic scale using a large-sample Poisson approximation, with SE[log(RR)]= $\sqrt{\frac{1}{a} + \frac{1}{c}}$, where where a is the count in the comparison period, c is the count in the pre-pandemic reference period, and SE is standard error. For hospital days, these RRs were interpreted strictly as aggregate bed-day volume and service-use indicators based on period-level totals per population person-years. Individual hospital days were not analyzed as separate independent patient-level observations. Because hospital days are clustered within patients and may be overdispersed, the simple count-based RRs were used as descriptive aggregate workload comparisons and were complemented by patient-level negative binomial regression models for total hospital days per patient.

Eligibility and attrition proportions were compared between periods using two-proportion comparisons. These included final eligible/initially identified patient proportions and final eligible/diagnostically confirmed proportions. Pearson chi-square tests were used to assess period-associated differences in categorical distributions, including disease-group distribution and eligibility-structure categories across the three study periods.

Continuous variables were summarized using medians and interquartile ranges, with means reported descriptively where informative. Because patient-level hospitalization variables were skewed, comparisons across the three study periods were performed using Kruskal-Wallis tests. These variables included age at first admission, admissions per patient, total hospital days per patient, and mean length of stay per patient. Sensitivity analyses were performed after stratification by major diagnostic group. Disease-specific RRs were calculated for final longitudinally eligible patients, admissions, and hospital days using population person-years as the denominator. Heterogeneity of disease-specific log-rate ratios was assessed using inverse-variance weighted Q statistics.

To distinguish aggregate service-use changes from patient-level utilization patterns, admissions per patient and hospital days per patient were modeled using negative binomial regression. These models were adjusted for period, major diagnostic group, sex, and age, and results were reported as incidence RRs (IRRs) with 95% CIs. Period-by-diagnostic-group interaction terms were explored in these models. Final longitudinal eligibility among diagnostically confirmed patients was examined using logistic regression adjusted for period and major diagnostic group, with results reported as odds ratios (ORs) and 95% CIs.

All statistical tests were two-sided, and p < 0.05 was considered statistically significant. Statistically significant p-values were marked in the tables. Because the study evaluated multiple case-capture, attrition, and service-use indicators, no formal correction for multiple comparisons was applied, and secondary analyses were interpreted as exploratory.

The study was reported in accordance with the STROBE (Strengthening the Reporting of OBservational Studies in Epidemiology) recommendations for observational studies [15]. Statistical analyses were performed using IBM SPSS Statistics for Windows, version 26 (released 2018; IBM Corp., Armonk, New York, United States), GraphPad Prism version 8.0.1 (Boston, Massachusetts, United States), and Microsoft Excel 2016 (Microsoft Corporation, Redmond, Washington, United States). Graphical outputs were prepared using GraphPad Prism and Microsoft Excel.

Ethics and data protection

The study was conducted in accordance with the Declaration of Helsinki and applicable European and Romanian data protection requirements. Data were deidentified before analysis. Ethical approval was granted by the Ethics Committee of Lucian Blaga University of Sibiu (approval number: 26/12.04.2023) and by the Ethics Committee of the Sibiu County Emergency Clinical Hospital (approval number: 13165/29.05.2023). Because the study was retrospective, non-interventional, and based on deidentified data, informed consent was waived according to the local ethical approvals.

## Results

Initially identified patients, diagnostic confirmation, and final eligibility

**Figure 1:**
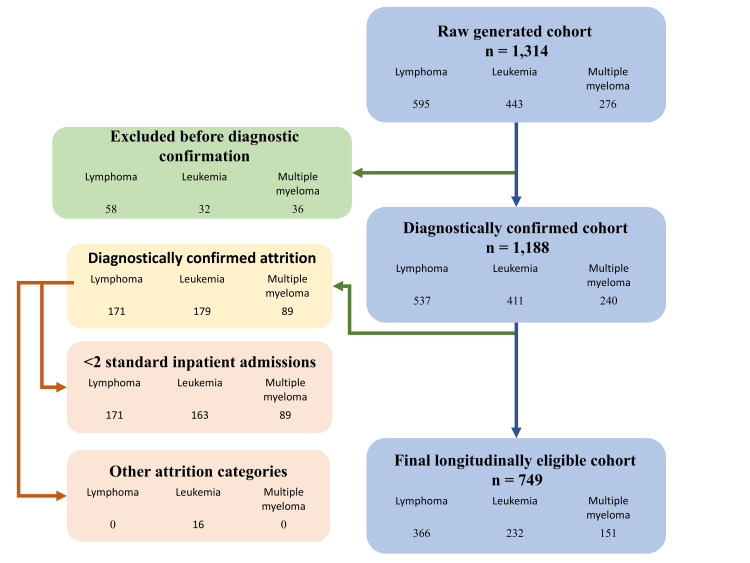
Cohort construction, diagnostic confirmation, and disease-group composition The figure shows the transition from initially identified patients to diagnostically confirmed cases and the final longitudinally eligible cohort, including disease-group counts and diagnostically confirmed exclusions. Image Credit: Author; created using Microsoft PowerPoint 2016 (Microsoft Corporation, Redmond, Washington, United States)

The initially identified patient cohort included 1,314 unique patients across the six-year interval. Of these, 1,188 patients were diagnostically confirmed as having hematologic malignancy, and 749 met the criteria for final longitudinal eligibility. Initially identified patients numbered 453 before the pandemic, 414 during the pandemic period, and 447 after restrictions. The corresponding numbers were 403, 367, and 418, respectively, for diagnostically confirmed patients, and 265, 229, and 255, respectively, for final longitudinally eligible patients. The raw-to-final cohort pathway, including diagnostic confirmation, diagnostically confirmed exclusions, and disease-group composition at each analytical layer, is summarized in Figure 1.

After population normalization, initially identified patients decreased during the pandemic period compared with the pre-pandemic period, although the difference was not statistically significant (RR 0.91, 95% CI 0.80-1.04, p = 0.176). Similar patterns were observed for diagnostically confirmed patients (RR 0.91, 95% CI 0.79-1.05, p = 0.186) and final longitudinally eligible patients (RR 0.86, 95% CI 0.72-1.03, p = 0.101). Period-level counts, eligibility proportions, and rate-ratio comparisons are shown in Table 1.

**Table 1:** Period-level case capture, eligibility, and inpatient service-use indicators. For case-capture rows, percentages indicate the distribution of each indicator across the three study periods. For hospitalizations and hospital days, percentages indicate the distribution of all hospitalization events and all hospital days across the three study periods. For eligibility-structure rows, percentages were calculated using the denominator specified in each indicator row. For “final eligible among initially identified patients,” initially identified patients from the same period were used as denominator. For “final eligible among diagnostically confirmed patients,” diagnostically confirmed patients from the same period were used as denominator. For count-based indicators, estimates are rate ratios using the pre-pandemic period as reference. For eligibility-structure indicators, estimates are absolute percentage-point differences. A p value <0.05 was considered statistically significant. *Statistically significant RR: rate ratio; pp: percentage points; CI: confidence interval; NA: not applicable

Indicator		Pre-pandemic	Pandemic	Post-restriction	Pandemic vs. pre-pandemic			Post-restriction vs. pre-pandemic		
					RR/pp	95% CI	p value	RR/pp	95% CI	p value
Case capture, n (%)	Initially identified patients	453 (34.5%)	414 (31.5%)	447 (34.0%)	0.91	0.80-1.04	0.176	0.99	0.87-1.13	0.85
	Diagnostically confirmed patients	403 (33.9%)	367 (30.9%)	418 (35.2%)	0.91	0.79-1.05	0.186	1.04	0.91-1.19	0.593
	Final longitudinally eligible patients	265 (35.4%)	229 (30.6%)	255 (34.0%)	0.86	0.72-1.03	0.101	0.96	0.81-1.14	0.667
Inpatient service use, n (%)	Hospitalizations	1,450 (34.3%)	1,325 (31.3%)	1,453 (34.4%)	0.91	0.85-0.98	0.015*	1	0.93-1.08	0.94
	Total hospital days	8,123 (36.9%)	6,553 (29.8%)	7,343 (33.3%)	0.81	0.78-0.83	<0.001*	0.90	0.88-0.93	<0.001*
Eligibility structure, n (%)	Final eligible among initially identified patients	265 (58.5%)	229 (55.3%)	255 (57.0%)	-3.2 pp	NA	0.344	-1.5 pp	NA	0.659
	Final eligible among diagnostically confirmed patients	265 (65.8%)	229 (62.4%)	255 (61.0%)	-3.4 pp	NA	0.332	-4.8 pp	NA	0.158

Inpatient service use and hospital day burden

Hospitalizations decreased from 1,450 before the pandemic to 1,325 during the pandemic period, corresponding to a significant reduction in the population-normalized hospitalization rate (RR 0.91, 95% CI 0.85-0.98, p = 0.015). After restrictions, hospitalizations increased to 1,453 and returned to the pre-pandemic level (RR 1.00, 95% CI 0.93-1.08, p = 0.940).

**Figure 2:**
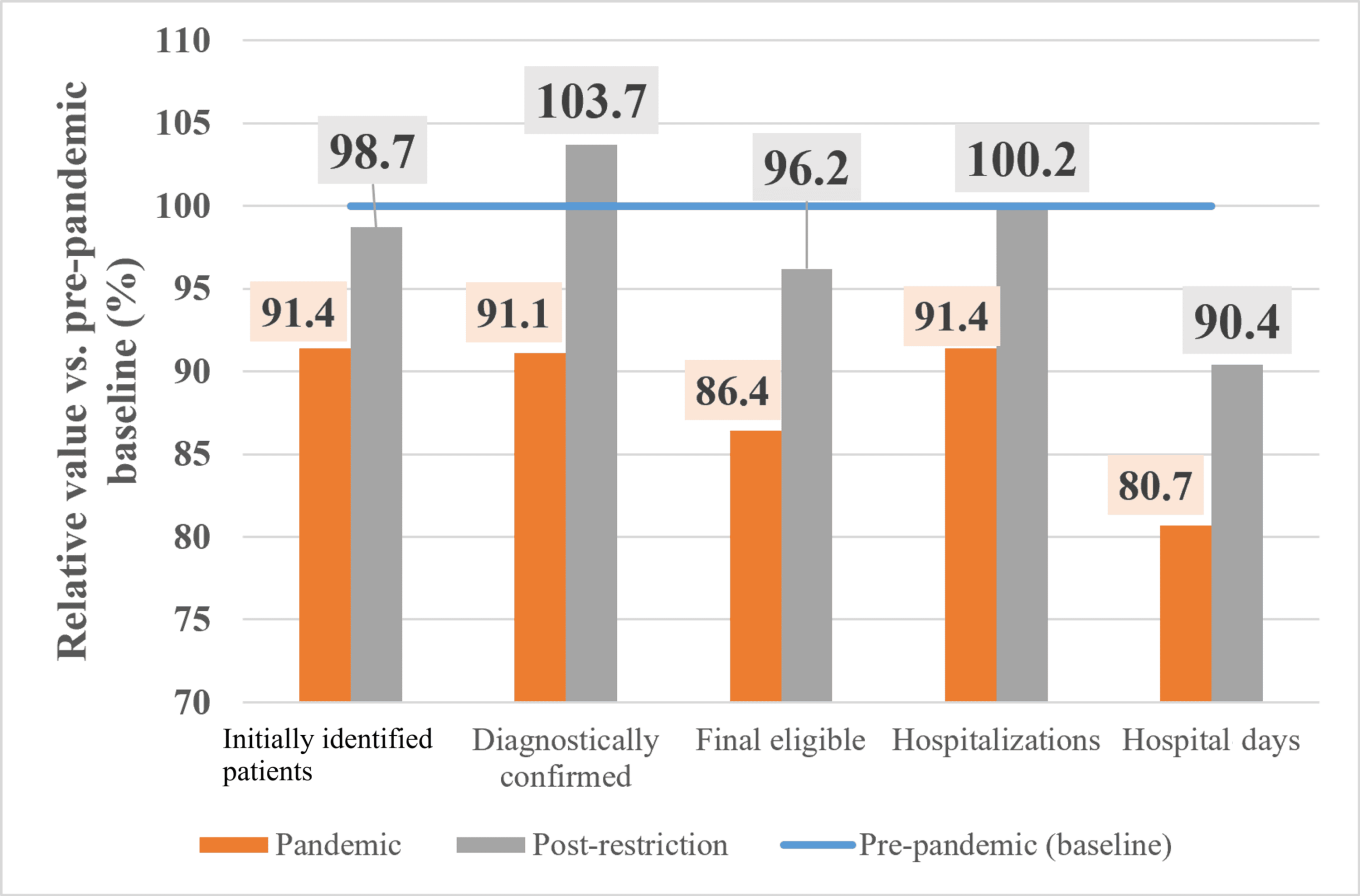
Relative changes in case-capture and inpatient service-use indicators across study periods Pre-pandemic values were set as 100%, and pandemic and post-restriction values are shown relative to the pre-pandemic baseline. Image Credit: Author; created using Microsoft Excel 2016 (Microsoft Corporation, Redmond, Washington, United States)

Total hospital days decreased from 8,123 before the pandemic to 6,553 during the pandemic period. The population-normalized hospital day rate decreased from 867.51 to 698.48 per 100,000 person-years, corresponding to an RR of 0.81 (95% CI 0.78-0.83, p < 0.001). After restrictions, total hospital days increased to 7,343, but remained below the pre-pandemic level (RR 0.90, 95% CI 0.88-0.93, p < 0.001). Relative changes in case-capture and inpatient service-use indicators are shown in Figure 2, while detailed population-normalized rates and rate-ratio comparisons are presented in Table 2.

**Table 2:** Population-normalized case-capture and inpatient service-use rates across study periods Rates are expressed per 100,000 person-years and represent hospital-based population-normalized case-capture and service-use indicators, not true population-based incidence estimates. RR comparisons use the pre-pandemic period as reference. A p value <0.05 was considered statistically significant. *Statistically significant PY: person-years; RR: rate ratio; CI: confidence interval

Indicator	Period	Count, n	Rate per 100,000 PY	RR	95% CI	p value
Initially identified patients	Pre-pandemic	453	48.38	-	-	-
	Pandemic	414	44.13	0.91	0.80-1.04	0.176
	Post-restriction	447	47.77	0.99	0.87-1.13	0.85
Diagnostically confirmed patients	Pre-pandemic	403	43.04	-	-	-
	Pandemic	367	39.12	0.91	0.79-1.05	0.186
	Post-restriction	418	44.67	1.04	0.91-1.19	0.593
Final longitudinally eligible patients	Pre-pandemic	265	28.3	-	-	-
	Pandemic	229	24.41	0.86	0.72-1.03	0.101
	Post-restriction	255	27.25	0.96	0.81-1.14	0.667
Hospitalizations	Pre-pandemic	1,450	154.85	-	-	-
	Pandemic	1,325	141.23	0.91	0.85-0.98	0.015*
	Post-restriction	1,453	155.29	1	0.93-1.08	0.940
Total hospital days	Pre-pandemic	8,123	867.51	-	-	-
	Pandemic	6,553	698.48	0.81	0.78-0.83	<0.001*
	Post-restriction	7,343	784.79	0.90	0.88-0.93	<0.001*

Patient-level measures were broadly comparable across periods. Median age at first admission was 65 years before the pandemic, 64 years during the pandemic period, and 66 years after restrictions (p = 0.166). Median admissions per patient were 4, 5, and 5, respectively (p = 0.207). Median total hospital days per patient were 17, 18, and 19, respectively (p = 0.919), while median mean length of stay per patient was 3.83, 3.50, and 3.53 days, respectively (p = 0.579). This indicates that the aggregate hospital day reduction was not accompanied by marked shifts in patient-level medians.

Adjusted negative binomial models, restricted to patients with repeated local inpatient representation in the final longitudinal cohort, showed comparable inpatient utilization within this retained inpatient cohort. These models used the patient as the unit of analysis and modeled admissions per patient and total hospital days per patient, thereby complementing the aggregate count-based RRs without treating individual hospital days as independent observations. After adjustment for major disease group, sex, and age, the pandemic period was associated with similar admissions per patient (IRR 1.06, 95% CI 0.95-1.18, p = 0.320) and hospital days per patient (IRR 0.94, 95% CI 0.81-1.10, p = 0.463). Period-by-disease-group interaction terms were also non-significant for admissions per patient (p = 0.787) and hospital days per patient (p = 0.773). These adjusted patient-level findings should therefore be interpreted as applying to patients retained in the final longitudinal cohort and not to all diagnostically confirmed patients.

Disease-group distribution and disease-specific rates

The final longitudinally eligible cohort included 366 lymphoma cases, 232 leukemia cases, and 151 multiple myeloma cases. Disease-group distribution in the final eligible cohort was similar across periods (chi-square = 1.46, df = 4, p = 0.833). Stable disease-group distributions were also observed in the initially identified patient cohort (chi-square = 0.32, df = 4, p = 0.988) and in the diagnostically confirmed cohort (chi-square = 0.85, df = 4, p = 0.931).

Disease-specific final eligible counts and rates are shown in Table 3. Lymphoma cases numbered 126 before the pandemic, 113 during the pandemic period, and 127 after restrictions (pandemic vs. pre-pandemic: RR 0.90, 95% CI 0.69-1.15, p = 0.392). Multiple myeloma cases numbered 50, 49, and 52, respectively (RR 0.98, 95% CI 0.66-1.45, p = 0.912). Leukemia cases numbered 89, 67, and 76, respectively, showing the largest numerical pandemic-period decrease among the three disease groups (RR 0.75, 95% CI 0.55-1.03, p = 0.077). None of the disease-specific final eligible comparisons reached statistical significance.

**Table 3:** Disease-specific final eligible rates per 100,000 person-years and period comparisons Values are shown as n (%) and hospital-based rates per 100,000 person-years in separate columns. These rates represent population-normalized hospital case-capture indicators for the final longitudinally eligible cohort and should not be interpreted as true disease-specific population incidence rates. Percentages were calculated using the total number of final longitudinally eligible patients in each study period as denominator: 265 patients in the pre-pandemic period, 229 patients in the pandemic period, and 255 patients in the post-restriction period. RR comparisons use the pre-pandemic period as reference. A p value <0.05 was considered statistically significant. No p values in this table reached statistical significance. RR: rate ratio; CI: confidence interval; PY: person-years

Disease group	Pre-pandemic		Pandemic		Post-restriction		Pandemic vs. pre-pandemic			Post-restriction vs. pre-pandemic		
	n (%)	Rate per 100,000 PY	n (%)	Rate per 100,000 PY	n (%)	Rate per 100,000 PY	RR	95% CI	p value	RR	95% CI	p value
Lymphoma	126 (47.5%)	13.46	113 (49.3%)	12.04	127 (49.8%)	13.57	0.9	0.69-1.15	0.392	1.01	0.79-1.29	0.945
Leukemia	89 (33.6%)	9.50	67 (29.3%)	7.14	76 (29.8%)	8.12	0.75	0.55-1.03	0.077	0.85	0.63-1.16	0.314
Multiple myeloma	50 (18.9%)	5.34	49 (21.4%)	5.22	52 (20.4%)	5.56	0.98	0.66-1.45	0.912	1.04	0.71-1.53	0.840

Disease-group-stratified service-use analyses showed significant pandemic-period reductions in aggregate hospital days across all three major groups: lymphoma (RR 0.83, 95% CI 0.79-0.87, p < 0.001), leukemia (RR 0.78, 95% CI 0.74-0.82, p < 0.001), and multiple myeloma (RR 0.80, 95% CI 0.73-0.87, p < 0.001). The heterogeneity test for pandemic versus pre-pandemic hospital day RRs was not significant (Q = 3.23, p = 0.198), indicating a broadly distributed bed-day reduction. Admission RRs were less uniform, with a significant reduction in leukemia admissions and no statistically significant change in lymphoma or myeloma admissions; heterogeneity across disease groups was significant for admissions (Q = 15.55, p = 0.00042).

Diagnostically confirmed attrition and exclusion structure

**Figure 3:**
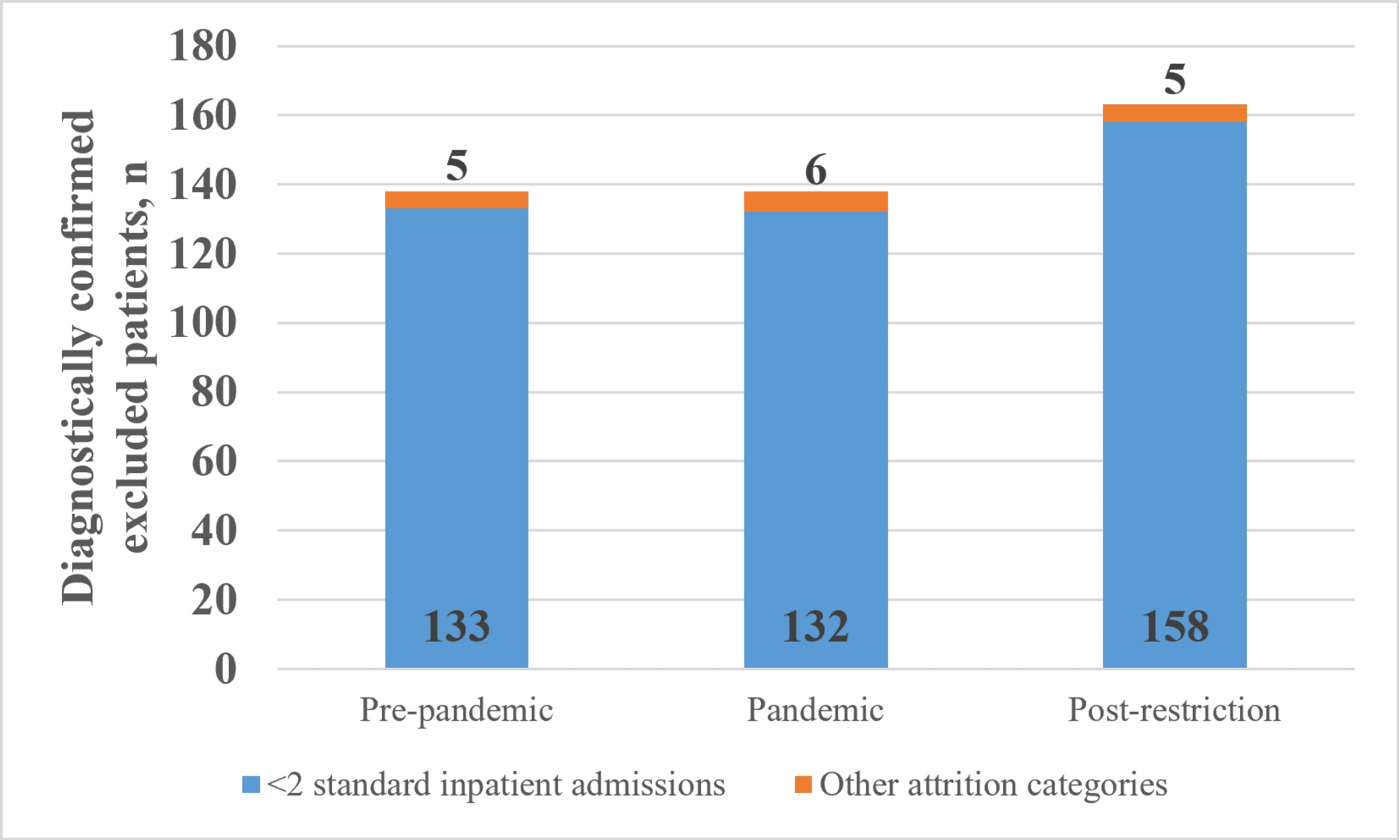
Diagnostically confirmed exclusions from the final longitudinal cohort by study period and exclusion category Bars show diagnostically confirmed patients excluded from the final longitudinal cohort, stratified by fewer than two standard inpatient admissions and other attrition categories. Image Credit: Author; created using Microsoft Excel 2016 (Microsoft Corporation, Redmond, Washington, United States)

Across the raw-to-final pathway, 565 initially identified patients were excluded from the final longitudinal cohort. Of these, 439 were diagnostically confirmed as having hematologic malignancy. The most frequent reason for diagnostically confirmed exclusion was fewer than two standard inpatient admissions, accounting for 423 cases. The remaining diagnostically confirmed exclusions were related to transfer outside the local hospital pathway or other exclusion reasons. Diagnostically confirmed attrition by study period and exclusion category is shown in Figure 3.

The final eligible/initially identified patient proportion was 58.5% before the pandemic, 55.3% during the pandemic period, and 57.0% after restrictions, with no statistically significant period difference. The final eligible/diagnostically confirmed proportion was 65.8%, 62.4%, and 61.0%, respectively, also without a statistically significant period effect. The diagnostically confirmed/initially identified patient proportion increased after restrictions compared with the pre-pandemic period, from 89.0% to 93.5% (+4.5 percentage points, 95% CI 0.9-8.2, p = 0.016; omnibus chi-square = 7.54, df = 2, p = 0.023).

Disease-specific, diagnostically confirmed exclusions are summarized in Table 4. Diagnostically confirmed exclusions were recorded in all three disease groups: 171 in lymphoma, 179 in leukemia, and 89 in multiple myeloma. The disease mix of diagnostically confirmed excluded patients was similar across periods (chi-square = 3.54, df = 4, p = 0.472).

**Table 4:** Disease-specific raw-to-final attrition by study period. Values are shown as n (%). Percentages for initially identified patients indicate the distribution of disease-specific initially identified patients across the three study periods. Percentages for diagnostically confirmed patients were calculated using initially identified patients from the same disease group and period as denominator. Percentages for final eligible and diagnostically confirmed excluded patients were calculated using diagnostically confirmed patients from the same disease group and period as denominator. Percentages for excluded patients with fewer than two admissions were calculated using diagnostically confirmed excluded patients from the same disease group and period as denominator.

Disease group	Period	Initially identified, n (%)	Diagnostically confirmed, n (%)	Final eligible, n (%)	Diagnostically confirmed excluded, n (%)	Excluded with <2 admissions, n (%)
Lymphoma	Pre-pandemic	207 (34.8%)	187 (90.3%)	126 (67.4%)	61 (32.6%)	61 (100.0%)
	Pandemic	184 (30.9%)	160 (87.0%)	113 (70.6%)	47 (29.4%)	47 (100.0%)
	Post-restriction	204 (34.3%)	190 (93.1%)	127 (66.8%)	63 (33.2%)	63 (100.0%)
Leukemia	Pre-pandemic	151 (34.1%)	138 (91.4%)	89 (64.5%)	49 (35.5%)	44 (89.8%)
	Pandemic	140 (31.6%)	128 (91.4%)	67 (52.3%)	61 (47.7%)	55 (90.2%)
	Post-restriction	152 (34.3%)	145 (95.4%)	76 (52.4%)	69 (47.6%)	64 (92.8%)
Multiple myeloma	Pre-pandemic	95 (34.4%)	78 (82.1%)	50 (64.1%)	28 (35.9%)	28 (100.0%)
	Pandemic	90 (32.6%)	79 (87.8%)	49 (62.0%)	30 (38.0%)	30 (100.0%)
	Post-restriction	91 (33.0%)	83 (91.2%)	52 (62.7%)	31 (37.3%)	31 (100.0%)

Adjusted logistic regression among diagnostically confirmed patients showed comparable final longitudinal eligibility across study periods. Relative to the pre-pandemic period, final eligibility was similar during the pandemic period (OR 0.87, 95% CI 0.65-1.17, p = 0.356) and after restrictions (OR 0.82, 95% CI 0.61-1.09, p = 0.162). Compared with lymphoma, leukemia showed lower adjusted odds of final eligibility (OR 0.61, 95% CI 0.46-0.79, p < 0.001), consistent with the larger leukemia attrition observed in the raw-to-final pathway.

## Discussion

The present study analyzed pandemic-period changes in hospital-based case capture, diagnostic confirmation, longitudinal inpatient representation, admissions, and hospital days among adults with hematologic malignancies treated in a Romanian tertiary center. The main finding was a measurable contraction in inpatient hematology service use during the pandemic period, driven primarily by fewer aggregate hospital days, while the broad lymphoma-leukemia-multiple myeloma distribution remained stable. This pattern places the study in a healthcare services framework, where patient counts, repeated inpatient representation, admissions, and bed-day volume are interpreted as connected indicators of service activity.

The sensitivity analyses support this interpretation. Disease-specific hospital day RRs showed significant pandemic-period reductions across lymphoma, leukemia, and multiple myeloma, without significant heterogeneity across groups. In contrast, admission RRs were less uniform, with a stronger leukemia-specific contraction. Adjusted patient-level models, restricted to patients retained in the final longitudinal cohort, showed comparable admissions and hospital days per retained patient across periods. Taken together, these findings suggest that the main pandemic-period signal was concentrated at the aggregate service-use level, rather than in a major change in per-patient inpatient utilization among patients with repeated local inpatient representation.

All-cancer registry studies provide a broader reference for pandemic-related diagnostic disruption. In Belgium, Peacock et al. reported a 44% reduction in invasive cancer diagnoses in April 2020 compared with April 2019, followed by incomplete recovery across 2020 [16]. In the Nordic countries, Johansson et al. analyzed 340,675 cancers diagnosed in 2020-2021 and reported first-wave incidence declines ranging from -21.7% in Sweden to -7.9% in Iceland, with smaller declines in later waves [17]. At the surveillance level, the Surveillance, Epidemiology, and End Results (SEER) Program reported a 10% decrease in all-cancer incidence in 2020 relative to 2019, followed by partial recovery in 2021 [18]. Irish registry data provide a further surveillance comparator. In the 2021 National Cancer Registry of Ireland (NCRI) preliminary analysis, microscopically verified cases remained below projected levels for several cancer sites; among cancers affecting both sexes, leukemia showed one of the largest total relative shortfalls (-33%), together with kidney cancer (-29%) and liver cancer (-25%). In sex-specific analyses, leukemia showed the largest relative shortfall among males (-40%), while male non-Hodgkin lymphoma and leukemia were below the projected range, and male multiple myeloma remained within the expected range [19].

Regional registry and pathology-based cohorts provide additional context for Central and Western European cancer care disruption. In Greater Poland, Trojanowski et al. reported 14,770 observed cancer diagnoses in 2020 compared with 18,154 expected diagnoses, with standardized incidence ratios of 0.80 in males and 0.83 in females [20]. In Northern Ireland, Hamilton et al. reported approximately 23% fewer pathological cancer diagnoses between March and September 2020 than expected [21]. In Málaga, Ruiz-Medina et al. reported fewer new cancer diagnoses during the first pandemic year than in 2019, although hematological tumors did not follow the general downward pattern [22], indicating that pandemic-related diagnostic disruption was substantial but not uniform across tumor groups, countries, and healthcare pathways.

Hematologic malignancies may therefore require a more pathway-specific interpretation than screening-dependent solid tumors. In Bavaria, Voigtländer et al. found a 6.7% reduction in malignant neoplasms reported by pathology departments during the pandemic, while non-Hodgkin lymphoma did not show a significant reduction [23]. In the Registro de Tumores de Madrid (Madrid Tumor Registry), Garrido-Cantero et al. reported moderate decreases in lymphoid and myeloid neoplasms in 2020, followed by near recovery in 2021 [24]. The Swedish Lymphoma Register provides a lymphoma-specific comparator, with fewer lymphoma diagnoses during March-June 2020 but annual incidence rate ratios close to the 2017-2019 reference period in 2020 and 2021 [8].

Specialist hematology service studies align more closely with the hospital-based perspective of the present analysis. Willan et al. reported 54% fewer new hematological malignancies diagnosed by histopathology during lockdown in Oxford, United Kingdom, with reductions across several hematologic categories [9]. Although their endpoint was diagnostic throughput rather than inpatient service use, both approaches capture how hematologic malignancy care moved through specialist hospital pathways under pandemic pressure. Larger hematology-specific COVID-19 cohorts also illustrate the clinical background of this pressure. The 2020 EPICOVIDEHA (Epidemiology of COVID-19 infection in patients with hematological malignancies: a European Haematology Association Survey) included 3,801 adult patients with hematologic malignancies and laboratory-confirmed COVID-19, of whom 73.1% were hospitalized, 18.1% required intensive care, and 31.2% died [25]. The subsequent 2020-2022 EPICOVIDEHA analysis included 8,767 cases from 152 centers in 41 countries and showed a fall in overall mortality across successive pandemic years [26].

Multiple myeloma studies provide disease-specific comparators for pandemic-related changes in diagnostic route, disease burden, and presentation pattern. Carmichael et al. analyzed 323 newly diagnosed myeloma patients across five English institutions and reported emergency-route diagnosis in 45.5% of post-COVID patients compared with 32.7% before COVID-19, together with higher frequencies of lytic and extramedullary disease [10]. Martinez-Lopez et al. used a global real-world data network and reported fewer new myeloma diagnoses in 2020 than in 2019, with delayed diagnosis and reduced survival among patients diagnosed in 2020 [11]. In the present cohort, final longitudinally eligible multiple myeloma counts remained stable across the three periods, at 50, 49, and 52 cases each, while aggregate hospital days decreased during the pandemic period and recovered after restrictions, suggesting that stable retained patient counts may coexist with changes in inpatient bed-day exposure.

Leukemia showed a different service-use profile. In the present cohort, leukemia had the largest numerical pandemic-period decrease in final longitudinally eligible patients and a significant reduction in admissions, while hospital days also declined significantly. Leukemia also showed lower adjusted odds of final longitudinal eligibility than lymphoma, consistent with the larger diagnostically confirmed attrition observed in the raw-to-final pathway. These findings do not define leukemia-specific clinical outcome differences, but they indicate that leukemia contributed meaningfully to the aggregate inpatient-service contraction. Leukemia-specific EPICOVIDEHA data provide clinical context for this pressure. Among 388 adult acute myeloid leukemia patients with COVID-19, severe or critical infection occurred in 62.3%, chemotherapy schedules were modified in 44.8%, and mortality reached 46.4% [27].

Hospital service studies from other settings further support the distinction between diagnosis counts and care-intensity indicators. Yerdenova et al. reported hemato-oncology service changes in Kazakhstan, including increased emergency admissions during pandemic years, higher mortality in 2020-2021 compared with adjacent periods, and more transfers from other hospitals [12]. In Manitoba, Decker et al. analyzed a general oncology system and found that new cancer diagnoses returned to baseline by August 2020, while radiotherapy fractions, urgent cancer-care visits, and in-person oncology visits remained reduced from April 2020 to June 2021 [28].

Disease-control and registry-based hematology data add further context to the vulnerability of hematologic malignancy services during the pandemic. The United Kingdom Coronavirus Cancer Monitoring Project cohort of 877 patients with hematologic malignancy and COVID-19 linked uncontrolled hematologic disease with higher mortality [29]. The Haematological Malignancy Research Network attributed 486 of 18,883 deaths in premalignant and malignant hematologic neoplasms to COVID-19 [30], while the Croatian KroHem cohort reported adverse outcomes among patients with lymphoid malignancies in the pre-Omicron period [31]. These outcome-oriented studies address different endpoints than hospital bed utilization, but they support the broader interpretation that hematologic malignancy care was exposed to both infection-related risk and service delivery pressure during the pandemic period. Against this background, the specific contribution of the present Romanian hospital-based cohort is the combined assessment of case generation, diagnostic confirmation, repeated inpatient representation, admissions, and hospital days, rather than another estimate of cancer incidence or disease-specific COVID-19 outcomes.

From a healthcare-services perspective, the present findings indicate that new diagnosis counts or final cohort size alone may not fully describe inpatient hemato-oncology service use during a disruption. A stable broad disease mix may coexist with fewer admissions, reduced aggregate bed-day exposure, or a larger group of diagnostically confirmed patients not represented in repeated inpatient care. A compact monitoring set combining raw case generation, diagnostic confirmation, final longitudinal eligibility, admissions, and hospital days may provide a descriptive framework for identifying whether changes occur mainly at the level of diagnostic volume, repeated local inpatient representation, or inpatient service intensity. Such indicators can be updated from routine hospital data and may support local descriptive monitoring when full staging, treatment response, survival, or patient-reported outcome data are not immediately available.

Limitations

This retrospective single-center study captured hospital case generation and standard inpatient use at the Sibiu County Emergency Clinical Hospital, but did not include outpatient visits, emergency-only encounters, referral delays, treatment modifications, chemotherapy delays, transfusion requirements, ICU admissions, mortality, survival, disease stage, treatment response, patient-reported and socioeconomic barriers to care, or ward-level capacity changes related to hospital repurposing during the pandemic. Therefore, the results describe hospital-based case capture and inpatient workload rather than the full clinical trajectory, treatment course, outcomes, access barriers, or institutional capacity constraints affecting patients with hematologic malignancies.

The requirement of at least two standard inpatient admissions may also have introduced selection bias by preferentially retaining patients with repeated local inpatient representation and excluding clinically relevant patients who had only one admission, were managed mainly in outpatient settings, were transferred early, continued care elsewhere, or died after a single hospital episode. For the same reason, adjusted patient-level analyses of admissions and hospital days apply only to the retained final longitudinal cohort and may not reflect utilization patterns among all diagnostically confirmed patients. Because inpatient episodes crossing a period boundary were allocated according to admission date, a small degree of boundary misclassification of hospital days is possible around March 11, 2020, and March 9, 2022.

Analyses were performed at the broad lymphoma-leukemia-multiple myeloma level because the objective was to evaluate service-use patterns across major hematologic malignancy groups. Subtype-level interpretation remained descriptive because several diagnostic categories contained small numbers. Population-normalized rates were interpreted as hospital-based case-capture and service-use indicators, not as population-incidence estimates. Diagnostically confirmed attrition was retained to distinguish confirmed cases without repeated local inpatient representation from patients included in the final longitudinal service-use cohort. The study compared three administratively defined two-year periods and did not use interrupted time-series or secular-trend modeling; therefore, the findings should be interpreted as descriptive period-level associations rather than causal estimates of the pandemic effect.

## Conclusions

This Romanian tertiary center cohort jointly assessed case capture, diagnostic confirmation, longitudinal inpatient representation, admissions, and hospital days in patients with hematologic malignancies across pre-pandemic, pandemic, and post-restriction periods. The pandemic period was associated with lower inpatient hematology service use, driven mainly by fewer aggregate hospital days, while broad disease group composition remained stable. Diagnostically confirmed attrition identified a substantial group of confirmed hematologic malignancy cases outside the final longitudinal inpatient cohort, most often because fewer than two standard admissions were recorded.

These findings should be interpreted as hospital-based service-use indicators rather than measures of full clinical trajectory, mortality, survival, treatment modification, or institutional capacity change. From a hospital-monitoring perspective, the combined assessment of case capture, diagnostic confirmation, longitudinal inpatient representation, admissions, and hospital days may serve as a descriptive indicator set for identifying whether changes occur mainly at the level of diagnostic volume, repeated local inpatient representation, or inpatient service intensity in this setting.
